# A novel label-free fluorescence assay for one-step sensitive detection of Hg^**2+**^ in environmental drinking water samples

**DOI:** 10.1038/srep45974

**Published:** 2017-04-05

**Authors:** Ya Li, Nan Liu, Hui Liu, Yu Wang, Yuwei Hao, Xinhua Ma, Xiaoli Li, Yapeng Huo, Jiahai Lu, Shuge Tang, Caiqin Wang, Yinhong Zhang, Zhixian Gao

**Affiliations:** 1 School of Public Health, Lanzhou University, Lanzhou 73000, P. R. China; 2 Tianjin Key Laboratory of Risk Assessment and Control Technology for Environment and Food Safety, Tianjin Institute of Health and Environmental Medicine, Tianjin, 300050, P. R. China; 3 School of Public Health, State Ministry of Education, Sun Yat-sen University, Guangzhou, Guangdong, 510080, P. R. China; 4 Department of Nutrition and Food Hygiene, College of Public Health, Zhengzhou University, Zhengzhou, 450001, P. R. China

## Abstract

A novel label-free fluorescence assay for detection of Hg^2+^ was developed based on the Hg^2+^-binding single-stranded DNA (ssDNA) and SYBR Green I (SG I). Differences from other assays, the designed rich-thymine (T) ssDNA probe without fluorescent labelling can be rapidly formed a T-Hg^2+^-T complex and folded into a stable hairpin structure in the presence of Hg^2+^ in environmental drinking water samples by facilitating fluorescence increase through intercalating with SG I in one-step. In the assay, the fluorescence signal can be directly obtained without additional incubation within 1 min. The dynamic quantitative working ranges was 5–1000 nM, the determination coefficients were satisfied by optimization of the reaction conditions. The lowest detection limit of Hg^2+^ was 3 nM which is well below the standard of U.S. Environmental Protection Agency. This method was highly specific for detecting of Hg^2+^ without being affected by other possible interfering ions from different background compositions of water samples. The recoveries of Hg^2+^ spiked in these samples were 95.05–103.51%. The proposed method is more viable, low-costing and simple for operation in field detection than the other methods with great potentials, such as emergency disposal, environmental monitoring, surveillance and supporting of ecological risk assessment and management.

Heavy metal ion-based pollution has become a critical global issue and a major source of human exposure stems from contaminated natural waters and potable water^1^,^2^,^3^. Particularly, mercury is a highly toxic heavy metal that severely threatens the human health^4^,^5^. Inorganic mercury, i.e., Hg and Hg^2+^ are released into environments through a variety of anthropogenic and natural sources. Industrial sources of mercury include coal and gold mining, solid waste incineration, wood pulping, fossil fuel combustion, and chemical manufacturing^6^,^7^,^8^. Additional sources of human exposure to mercury include the household^9^ and workplace^10^, religious practices^11^, dental amalgams^12^,^13^,^14^, and vaccines^15^,^16^. Furthermore, Hg^2+^ is not readily able to be biodegraded and is cline to be accumulated in organisms, which causes diverse human diseases such as kidney failure, prenatal brain damage, cognitive and motion disorders, vision and hearing loss, and even death^17^,^18^. It is also a neurotoxin and causes immune system dysfunction^9^,^19^.

Based on the serious harms of Hg^2+^ to human health and the green environments, it is quite necessary to develop a rapid and low-cost method for detection of Hg^2+^ with high sensitivity and selectivity. Conventional quantitative approaches for Hg^2+^ analysis in water samples include inductively coupled plasma mass spectrometry (ICP-MS)^19^, atomic fluorescence spectroscopy (AFS)^20^, atomic absorption spectroscopy^21^, fluorescent spectrophotometry^22^ and X-ray absorption spectroscopy^23^, etc. However, these methods usually involve sophisticated instruments, complicated sample preparation, large sample volume and professional training for operation, which limiting their widespread application. Therefore, the development of novel method for Hg^2+^ detection that are sensitive, selective, cost-effective, rapid, facile and applicable to the environmental and biological milieus is an important technological supporting for Hg^2+^ contamination control and disposition.

SYBR Green I (SG I) is acted as one of the fluorescent dyes, has become gradually significant for a wide range of analytic and diagnostic fields^24^,^25^. Since its introduction, SG I has been applied productively in the detection of nucleic acids in gels^26^, fluorescence imaging techniques^27^, real-time PCR^28^ and biochip applications^29^, etc. It tends to bind into the double-stranded DNA (dsDNA) and can be excited out bright fluorescence signal, neither of the single-stranded DNA (ssDNA) which only excited out weak fluorescence signal. The real formation of SG I is seldom reported, while the patent 5658751^30^ of United States explicates that, the core structure of SG I is on the basis of a monomeric unsymmetrical cyanine dye comprising an N-alkylated benzothiazolium or benzoxazolium ring system, which is bound by a monomethine bridge to a pyridinium or quinolinium ring system that carries a substituent with a heteroatom. The concrete concentration of 10,000× is very likely to be 10 mg mL^−1^ (19.6 mM)^31^. It reported that SG I exhibits fluorescence via surface or groove binding by comparing other dsDNA-specific dyes^32^, the other study showed that the fluorescence from SG I interaction with dsDNA depends on the ratio of base pair/SG I molecule complexes^33^. The exact mechanisms that how SG I linking/coupling to dsDNA still remains to be unknown. Ono’s group initially confirmed that Hg^2+^ can specifically interact with Ts to form a T-Hg^2+^-T complex for the designing of molecular beacon^34^.

According to the above principles, we designed a novel label-free fluorescence assay for one-step and rapid detection of Hg^2+^ in environmental drinking water samples by forming a stable hairpin structure of the Hg-binding ssDNA and the interaction of SG I and the ssDNA resulted in strong fluorescence in the present of Hg^2+^. The proposed method is sensitive, convenient, rapid and economic, and meet the requirements of field detection.

## Results

### Strategy for Hg^2+^ detection

**Figure 1:**
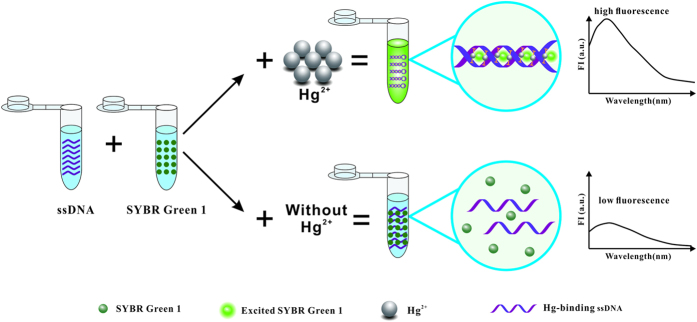
Schematic illustration of label-free fluorescence method for detection of Hg^2+^.

In this study, we designed a T-rich ssDNA i.e., the Hg-binding ssDNA which containing of 24 bases with seven T-T mismatches, guanine (G) or cytosine (C) base was in either end of the ssDNA. In the absence of Hg^2+^, the ssDNA adopted a random coil structure, there was only a weak fluorescence by the addition of SG I. By means of the computational molecular simulation for structure display and free energy determination by Mfold, ΔG_Hairpin loop_ = 2.98 KJ/mol, ΔG_External loop_ = −0.49 KJ/mol (Fig. S1). It suggests that the secondary construct of Hg-binding ssDNA is stable enough that the values of ΔGs are low^35^ which is not very likely to be formed in dsDNA by self-hybridization and not able to cause false positive results. It demonstrated in Fig. 1 that in the presence of Hg^2+^, hence the formation of T-Hg^2+^-T (Fig. S2), the ssDNA can be rapidly formed a T-Hg^2+^-T complex and folded into a stable hairpin structure with a dsDNA sequence in stem of the hairpin which is more stable than the Watson-Crick A-T pair^36^. When SG I is added, it embeds grooves of double helix structure which resulted in a strong fluorescence with emission at 530 nm. The intensity of fluorescence and the quantity of the dsDNA presents a positive correlation. And therefore, according to the recorded FI, it was able to speculate the concentration of Hg^2+^ in environmental drinking water samples (Fig. 1).

### Optimization of experimental conditions

**Figure 2:**
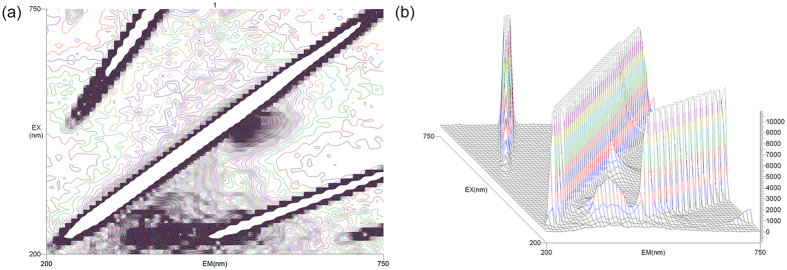
The 3D fluorescent scanning images of SG I. (**a**) The 3D fluorescent contour spectrogram. (**b**) The 3D fluorescent spectrogram.

In order to improve the detection performance, experimental conditions were optimized. As shown in Fig. 2, the area of optimum excitation wavelength and the scattered signal was recorded by 3D scanning. Confirmation of optimal excitation wavelength was based on the 3D fluorescent scanning images and the current report^31^. Thus, all the measurements were carried out with excitation at 490 nm and emission at 535 nm. The efficiency of the detection system would be enhanced greatly.

Figure S3 presented that the obtained FIs were progressively decreasing with time scanning. The reactants were mixed evenly and determined by F-4500 immediately within 1 min. Figure S4 displayed the effects of different intercalated amounts of SG I. When the addition of SG I was 2 μL (100×), it demonstrated the highest fluorescence enhancement. It is suggested that, in the optimized amount of SG I embedded in dsDNA, it was appropriately at the saturation point under this condition. The occurrence of fluorescence quenching was caused by accumulation of excessive 2 μL SG I probably owing to the steric hindrance of the reactions. However, the mechanism needs further research. In addition, the pH value of the buffer solution was 7.6 which is closed to the pH value of human body fluid. And the competitive coordination of OH^−^ with Hg^2+^ limits the formation of T-Hg^2+^-T structure in an alkaline solution^36^.

### Sensitivity of the novel one-step assay for Hg^2+^ detection

**Figure 3:**
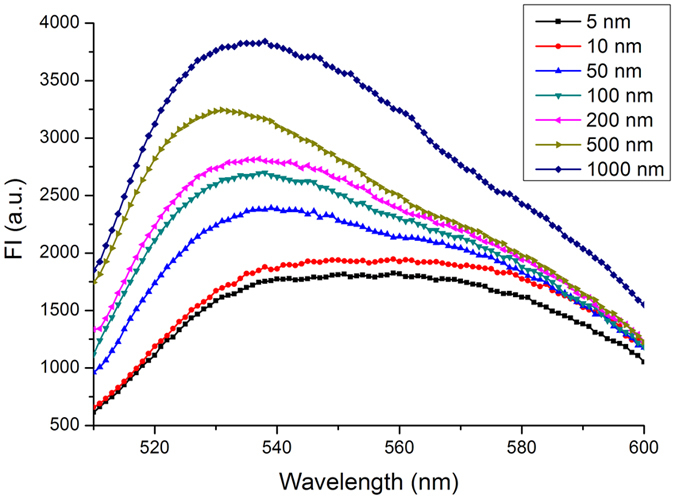
Fluorescence response of the test system at different Hg^2+^ concentrations.

**Figure 4:**
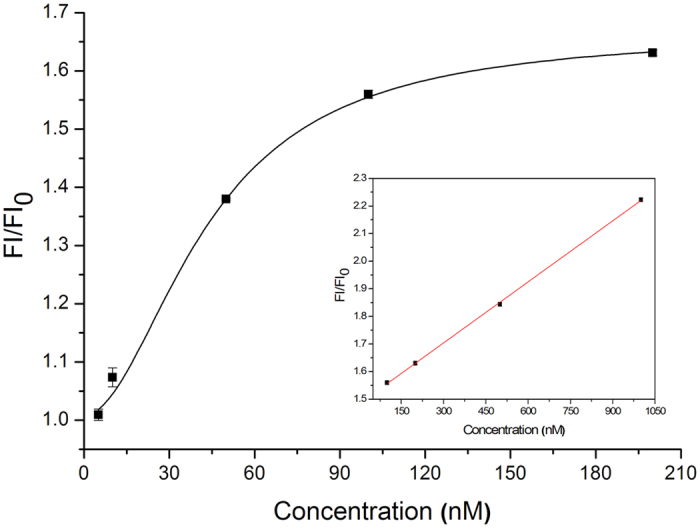
The standard curves for the detection of Hg^2+^ by the novel one-step assay. The logistic regression equation and the linear equation in the inset graph with different working ranges.

To assess the sensitivity and dynamic range of the proposed detection, a various of concentrations of Hg^2+^ were determined based on the records of the fluorescence signal. Figure 3 displayed that the fluorescence response of the test system at different Hg^2+^ concentrations positively increased. The logistic regression equation was y = 0.5625–0.6577/(1 + (x/43.5960)^1.9243^) with a determination coefficient (*R*^*2*^) of 0.9961 and the working range of 5–200 nM (Fig. 4); the linear equation assay was y = 7.3725x + 1.4825 (*R*^*2*^ = 0.9996) with the working range of 100–1000 nM in the inset graph of Fig. 4. Reasonably, the LDL of the developed approach for Hg^2+^ was 3 nM relied on 3σ which was lower than the standard of U.S. Environmental Protection Agency in drinking water (10 nM). σ is described as the standard deviation of the blank signal (without Hg^2+^ addition). Furthermore, the whole detection can be achieved within 1 min which proved that the proposed method is highly sensitive and rapid to meet the demand for the detection of Hg^2+^ especially in the on-site test.

### Specificity of the novel one-step assay for Hg^2+^ detection

**Figure 5:**
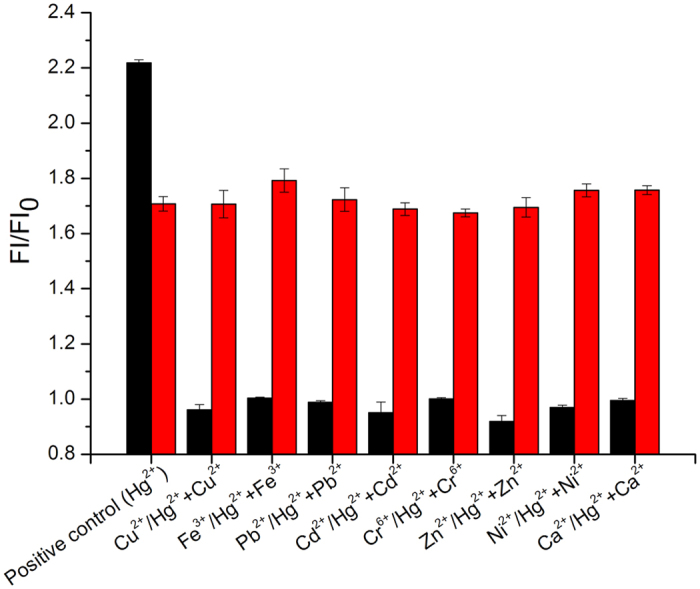
Selectivity of Hg^2+^ of the test system. Positive control: Black column: FI/FI_0_ of 1 μM Hg^2+^; Red column: FI/FI_0_ of 300 nM Hg^2+^. Other: Black column: FI/FI_0_ of 1 μM interfering metal ion; Red column: FI/FI_0_ of 900 nM interfering metal ion mixed with 300 nM Hg^2+^.

Specificity of detection system for Hg^2+^ was verified in two conditions. The first was that all the other concentration of the interfering ions were the same as the concentration of Hg^2+^ in the samples. The obtained values of FI/FI_0_ were illustrated in the black column of Fig. 5 and the two groups were significantly different. To further evaluate the practical applicability of analytical method for Hg^2+^, the positive control experiments were also carried out by adding 300 nM Hg^2+^ to the other interfering metal ion with the concentration of 900 nM (Trice higher than that of the sole Hg^2+^ addition), respectively. As indicating in the red column of Fig. 5, the obtained FIs were very closed to that of the addition of 300 nM Hg^2+^ only. The system appeared to be specific for Hg^2+^ detection with no significant cross-reactivity by the other metal ions. Both of the two conditions likewise suggested that interferences from diverse interfering metal ions having little effects on the detection system of Hg^2+^. It is accessible to satisfy the selectivity requirements of the Hg^2+^ detection in water environment.

### Practical appraisal of the detection platform

The practical application of the developed method was evaluated by the determination of the recovery of spiked Hg^2+^ in drinking water and ambient water samples, and compare to the classical laboratory assay, i.e. AFS, which is also the national standard of China for detecting Hg^2+^ in drinking water (GB/T-5750.6-2006). The analytical results were illustrated in Table 1. We observed that the recovery values were 95.05–103.51% by the developed method and 96.39–106.94% by AFS compared with the real values with the relative standard deviations below 10% which indicating good recovery in the assay. The proposed method and AFS gave consistent results. It suggested that the potential interference from the different background composition of water samples employed by this method was negligible and there were no significant differences between the two methods. Moreover, with the rapidness, convenience and low-costing of the proposed detection platform, it is applicable to analyze Hg^2+^ in practical environmental drinking water samples.

## Discussion and Conclusions

Compared with the previous works about Hg^2+^ detection based on DNA^37^,^38^,^39^,^40^,^41^,^42^,^43^,^44^ (Table 2) and/or rhodamine derivatives^45^,^46^, the proposed method is much simpler, the designed ssDNA probe doesn’t require fluorescent labelling and all the detection processes of Hg^2+^ can be accomplished at RT within 1 min in one step without additional incubation. As can be seen, the developed method avoids a time-consuming derivatization step, especially compared with surface-enhanced Raman spectroscopy^40^, chemiluminescence detection^44^ and fluorescent chemodosimeter based on the rhodamine spirolactam derivative^45^,^46^, etc. Besides the above, under the optimized conditions, this novel method presents higher sensitivity than that of the rhodamine-based method (97 nM, 100 nM)^45^,^47^.

In summary, we have developed a one-step straightforward and reliable T-rich DNA based fluorescence strategy for detecting of Hg^2+^ in environmental drinking water samples by designing a specific ssDNA probe and thus formation of a T-Hg^2+^-T complex in the presence of Hg^2+^ by folding to a stable hairpin structure. The assay also relies on the extraordinary fluorescence turn-off mechanism of SG I. The proposed method is more viable, low-costing and simple for operation in field detection than the other methods, making it easy to be integrated and automatized in microfluidic chip and sensors in the future. Great potentials are applicable by the proposed assay in detection of heavy metals for emergency disposal, environmental monitoring, surveillance and supporting of ecological risk assessment and management.

## Methods

### Chemicals and Materials

ssDNA was designed by Mfold (http://unafold.rna.albany.edu/?q=mfold/DNA-Folding-Form). They were commercially synthesized by Sangon Biotech Co., Ltd (Shanghai, China), and purified by high performance liquid chromatography. The sequences of the oligonucleotides were: 5′-CTTCTTTCTTCCCCTTGTTTGTTG-3′, hereinafter referred to “Hg-binding ssDNA”. SG I (10,000× concentrate dissolved in dimethyl sulfoxide) was ordered from Invitrogen Biotechnology Co., Ltd (Shanghai, China) and stored at −20 °C. The standard solutions of Hg^2+^, Pb^2+^, Ni^2+^, Zn^2+^, Ca^2+^, Cu^2+^, Fe^3+^, Cr^6+^ and Cd^2+^ were purchased from AccuStandard, Inc. (New Haven, USA) with the concentration of 1 mg mL^−1^ respectively. All the other chemicals were of analytical-reagent grade. Ultra-purified water used in the experiments was prepared by Milli-Q system (Millipore, Bedford, MA), which had a minimum resistivity of 18 MΩ·cm. All the experiments were performed at room temperature (RT).

### Apparatus

Fluorescence intensities (FIs) values were all recorded and analyzed by F-4500 fluorescence spectrophotometer (Hitachi, Tokyo, Japan) with the following parameters: a response time of 2 s, a PMT voltage of 700 V, a scan speed of 1200 nm min^−1^, an excitation wavelength of 490 nm, all the excitation and the emission slits were 5 nm. The laboratory method for the detection of Hg^2+^ was employed by AFS (GB/T-5750.6-2006, China) (AFS-9800, bjhaiguang, Beijing, China).

### Preparation of the Hg-binding ssDNA

The primitive commercialized lyophilized oligonucleotides powder in EP tube was centrifuged at 10000 rpm for 1 min and then dissolved with ultra-purified water to 100 μM. The stock solutions of Hg-binding ssDNA was prepared after gently vortex (600–800 rpm) and frozen at −20 °C. The desired concentration of the ssDNA was diluted with HEPES buffer solution (HEPES: 10 mM, NaNO_3_: 20 mM, pH: 7.6).

### Optimization of experimental conditions

The Hg-binding ssDNA was diluted to the concentration of 100 nM in microtubes, Hg^2+^ (1 μM) and SG I (2 μL, 100×) were added and gently vortexed for 3 min at RT. 3D scanning was operated in excitation and emission ranging from the wavelength of 200 to 750 nm. The 3D fluorescent spectrograms were obtained by Hitachi FL Solutions software. And then, various amounts of 2 μL SG I (400×, 200×, 100×, 40× and 20×) were prepared and were respectively added into the above mixed solution. Afterwards, with the same procedure, optimal amount of SG I was obtained in wavelength ranging from 510 to 600 nm with excitation at 490 nm. Then the addition amount of SG I is unchanged and time scanning were performed by fluorescence spectrophotometer.

### Assay for the determination of Hg^2+^

Under the optimal amount of SG I, the concentration gradients of the Hg^2+^ were added into the probe solutions of Hg-binding ssDNA and then the above prepared solutions were mixed subsequently at RT for 1 min. Fluorescence spectra were measured in wavelength ranging from 510 to 600 nm with the excitation wavelength at 490 nm. The FI at wavelength of 535 nm was recorded for quantitative assay of Hg^2+^. The process could be achieved in one step. All the measurements were repeated in triplicate.

### Specificity test

To verify the specificity of the system for determining Hg^2+^, we analyzed the potential interfered ions in real water sample, such as Hg^2+^, Pb^2+^, Ni^2+^, Zn^2+^, Ca^2+^, Cu^2+^, Fe^3+^, Cr^6+^ and Cd^2+^, etc. All the water samples were tested without the existence of Hg^2+^ before. Furthermore, the concentration of each metal ion in the experimental group and the concentration of Hg^2+^ as positive control was 1 μM. The other experimental group was carried out by adding 300 nM Hg^2+^ to the interfering metal ion with the concentration of 900 nM, and the positive control was 300 nM Hg^2+^ only.

### Preparation of practical water sample

To evaluate the practical application of the assay, drinking water samples including tap water from Tianjin (117°12′7″E, 39°06′41″N) and lake water from Hangzhou (119°0′35″E, 29°35′54″N) were employed for Hg^2+^ detection. To get rid of the potential inference of chlorine that might exist, the water samples were heated 5 min at 85 °C and let it stand for about 2 d, then stored at 4 °C for further use. All the water samples were filtered through 0.45 μm microfiltrators and detected by AFS. We found Hg^2+^ hasn’t been detected. After that, AFS was carried out for Hg^2+^ detection. After AFS detection, Hg^2+^ in the initial water samples hasn’t been detected yet. Therefore the extra addition of Hg^2+^ was spiked in these samples to verify there is no matrix effects as the interference factor for Hg^2+^ by our proposed method. The aforementioned water samples were used as a sample matrix to evaluate the recovery in the assay. Hg^2+^ was spiked into aforementioned water samples, and analyzed by the proposed method and AFS separately.

## Additional Information

**How to cite this article:** Li, Y. *et al*. A novel label-free fluorescence assay for one-step sensitive detection of Hg^2+^ in environmental drinking water samples. *Sci. Rep.* **7**, 45974; doi: 10.1038/srep45974 (2017).

**Publisher's note:** Springer Nature remains neutral with regard to jurisdictional claims in published maps and institutional affiliations.

## Supplementary Material

Supplementary Information

## Figures and Tables

**Table 1:** Recovery tests of Hg^2+^-spiked water samples by the proposed method and AFS (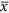 ± s, n = 3, nM).

Water samples	spiked	Proposed method	Recovery (%) of the proposed method	AFS	Recovery (%) of AFS
Drinking water 1	25	23.76 ± 1.27	95.05	24.85 ± 0.35	99.39
Drinking water 2	50	48.04 ± 1.09	96.07	52.15 ± 0.65	104.69
Drinking water 3	100	100.46 ± 2.44	100.46	107.20 ± 1.10	106.94
Drinking water 4	200	198.22 ± 2.45	99.11	192.05 ± 0.80	96.39
Lake water 1	25	25.88 ± 2.14	103.51	25.50 ± 0.40	101.66
Lake water 2	50	49.45 ± 1.57	98.90	51.55 ± 0.80	102.07
Lake water 3	100	99.82 ± 3.34	99.82	104.25 ± 0.85	104.48
Lake water 4	200	201.18 ± 1.94	100.59	215.80 ± 2.70	106.81

**Table 2:** Comparison of LDL, calibrated range and detection time, etc. among other DNA based methods for determination of Hg^2+^.

Detection method	LDL (nM)	Calibrated range (nM)	Reaction and incubation time	Operation	Ref
Electrochemiluminescence	0.2	0.5–1.0 × 10^3^	50 min	laborious	[38]
Field effect transistor	0.1	0.1–10	1–2 s	laborious	[39]
Surface-enhanced Raman spectroscopy	1.0 × 10^−3^	1.0 × 10^−3^–2.0 × 10^7^	>2 hr	moderate	[40]
UV-vis spectrophotometric	3.5	11.5–4.75 × 10^3^	10 min	laborious	[42]
Capillary electrophoresis	4–5	4–5.0 × 10^2^	40 min	laborious	[43]
Chemiluminescence	2	2–1.0 × 10^2^	>3 hr	moderate	[44]
T-Hg^2+^-T mis-matched fluorescent method	9.5	32–1.8 × 10^3^	30 min	convenient	[41]
Proposed fluorescent method	3	5–1.0 × 10^3^	1 min	convenient	this work
