# Supplementary material for: A novel label-free fluorescence assay for one-step sensitive detection of Hg2+ in environmental drinking water samples
Source: Sci Rep. 2017 Apr 5;7:45974. doi: 10.1038/srep45974 (PMC5380999; doi:10.1038/srep45974)
Supplement: Supplementary Information [file srep45974-s1.pdf]

Supplementary Information

A novel label-free fluorescence assay for one-step sensitive detection of Hg^{2+} in environmental drinking water samples

Ya Li[1, 2], Nan Liu[1, 2, 3, 4]*, Hui Liu[1], Yu Wang[1], Yuwei Hao[1], Xinhua Ma[2], Xiaoli Li[2], Yapeng Huo[2], Jiahai Lu[3], Shuge Tang[2, 4], Caiqin Wang[1, 2], Yinhong Zhang[1*] and Zhixian Gao[2]*

[1]School of Public Health, Lanzhou University, Lanzhou 73000, P. R. China

[2]Tianjin Key Laboratory of Risk Assessment and Control Technology for Environment and Food Safety, Tianjin Institute of Health and Environmental Medicine, Tianjin, 300050, P. R. China

[3]School of Public Health, State Ministry of Education, Sun Yat-sen University, Guangzhou, Guangdong, 510080, P. R. China

[4]Department of Nutrition and Food Hygiene, College of Public Health, Zhengzhou University, Zhengzhou, 450001, P. R. China

*Corresponding author: Y. Z. (email: zhangyh@lzu.edu.cn), N. L. (email: LNQ555@126.com), and Z. G. (email: gaozhx@163.com)

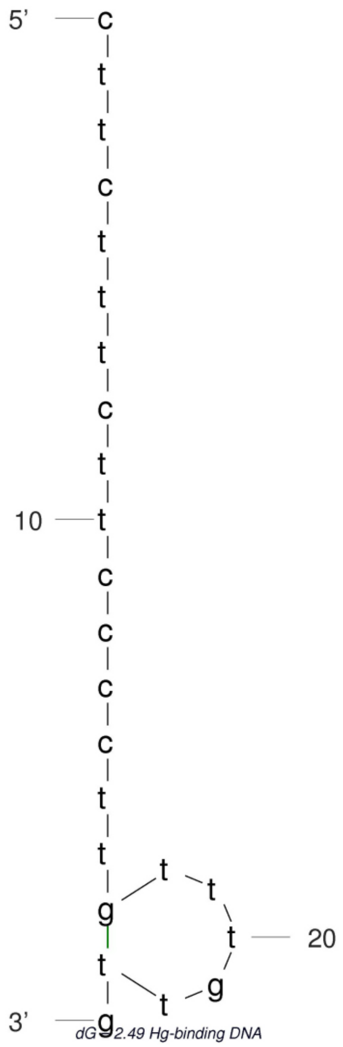

Fig. S1. The simulated image of ssDNA.

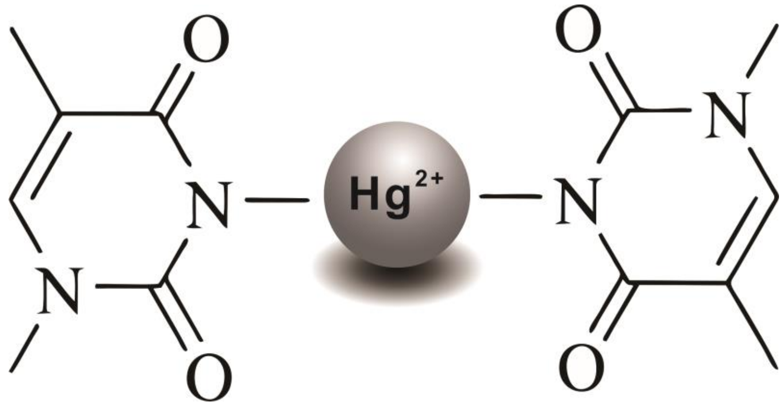

Fig. S2. The structure of T-Hg^{2+}-T.

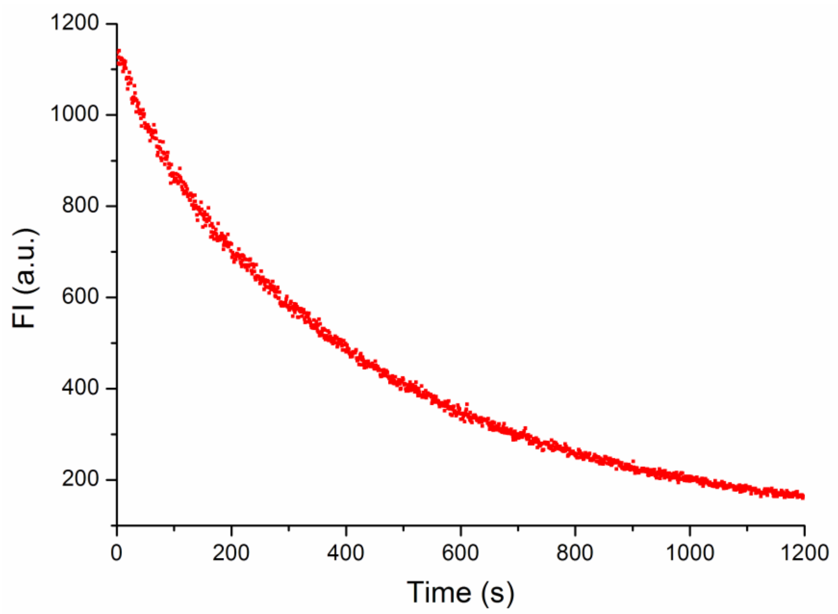

Fig. S3. Fluorescence response of the test system with the time scanning.

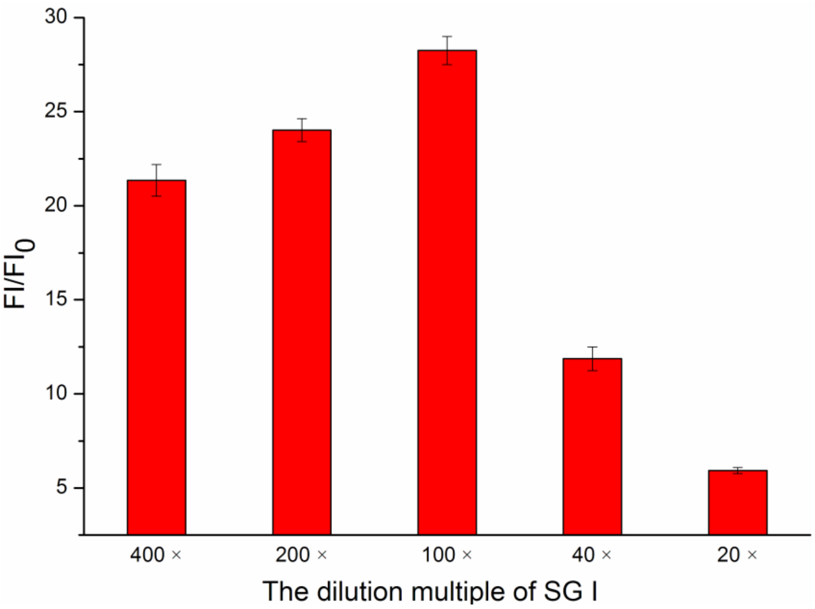

Fig. S4. Fluorescence response of the test system at different dilution multiple of SG I.
